# Unusual presentation of the germinoma: A 26-year-old female

**DOI:** 10.22088/cjim.12.0.397

**Published:** 2021

**Authors:** Zahra Davoudi, Arezoo Chouhdari, Adineh Taherkhani, Farahnaz Bidari Zerehpoosh, Mohammad Samadian

**Affiliations:** 1 Skull Base Research Center, Loghman Hakim Medical Center, Shahid Beheshti University of Medical Sciences, Tehran, Iran; 2 Department of Internal Medicine, Loghman Hakim Medical Center, Shahid Beheshti University of Medical Sciences, Tehran, Iran; 3 Department of Pathology, Shahid Beheshti University of Medical Sciences, Tehran, Iran

**Keywords:** Germinoma, Unusual, Presentation

## Abstract

**Background:**

Germinoma is a rare lesion found commonly in the pineal and suprasellar regions of the brain. Clinical presentation mainly involves the location and size of the tumor and the patient age. Endocrine abnormalities are the most common symptom.

**Case Presentation:**

The patient was a 26-year-old Iranian female who suffered from germinoma for a long time and was referred to Loghman Hakim Hospital for amenorrhea, polyuria, and polydipsia. Despite diagnostic challenges, she was finally diagnosed with suprasellar germinoma after endoscopic transsphenoidal surgery, followed by radiotherapy and medical interventions to complement the surgery.

**Conclusion:**

It is important to be able to diagnose the patient's problem at an early stage based on their history, hormonal profile, laboratory results and radiological view.

Areas around the third ventricle, especially in the pineal and suprasellar compartments, are the most common sites of intracranial germ cell tumors (GCTs), which involve both children and adults (1).CNS GCTs have been observed most commonly among young patients with approximately 90% of cases < 20 years. Its highest incidence rate was observed among patients between 10 and 12 years of age (2).The common symptoms of suprasellar germinomas are diabetes insipidus (DI), visual impairment, and hypothalamic-pituitary failure (3).More than 90% of the patients responded to chemotherapy (CT) and radiotherapy (RT), turning its early diagnosis into the most important part of an effective treatment (4).The reported case of germinoma was a patient who underwent a transsphenoidal surgery with a primary diagnosis of pituitary adenoma and clinical manifestations of pituitary dysfunction and DI. 

## Case Presentation

The case was a 26-year-old Iranian female presented to the endocrinology clinic of Loghman Hakim Hospital because of amenorrhea, polyuria, polydipsia, and morning nausea. She mentioned that her oligomenorrhea started in her first menstrual period at the age of 13 followed by amenorrhea after some time. She was diagnosed with polycystic ovarian disease (PCOD) and prescribed oral contraceptive agents (OCP) for 5 years. After the treatment period, the patient continued to suffer from menstruation disorders. Therefore, she underwent new tests ordered by the endocrinologist and the results were as follows: FSH: 0.4(normal range: 2-11) mIU/ml; LH: 0.3(normal range: 2-11) mIU/ml; PRL: 85 (normal range: 5.2-26.5) ng/ml; TSH: 0.1(normal range: 0.3-4.2) μIU/ml; and FT4: 12.7 (normal range: 15-23) ng/ml. Due to a high prolactin level, magnetic resonance imaging (MRI) of the brain was requested.

**Figure1:**
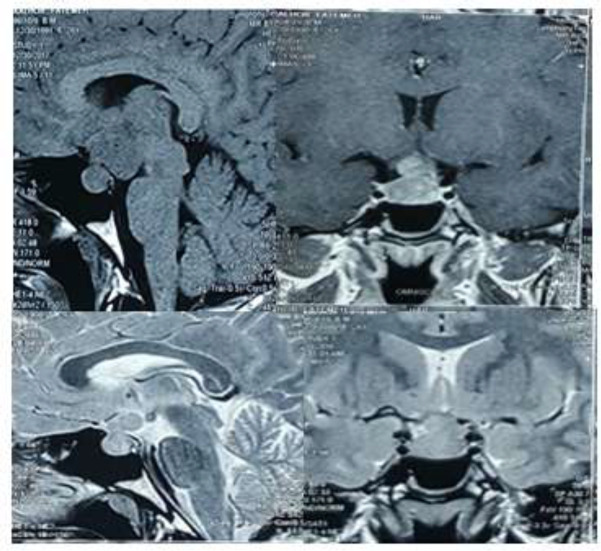
Multilobar sellar mass with heterogeneous enhancement and suprasellar growth, T1- and T2-weighted gadolinium-enhanced sagittal and coronal images

The MRI results showed a multilobar sellar mass (approximately, 30×27×24 mm^3^) with heterogeneous enhancement and suprasellar growth (figure 1). 

She was primarily misdiagnosed as macroprolactinoma and treated with cabergoline (dostinex) 0.5 mg twice per week. Despite at least 4-month treatment and prolactin normalization, not only menstruation was not managed but also the symptoms of polyuria, polydipsia, dry mouth, frontal headache behind the eyes, blurred vision, and morning nausea appeared. Therefore, dostinex 0.5 mg was discontinued and the patient was referred to the our endocrinology clinic by a neurosurgeon. Brain MRI, laboratory tests, and DI assessment tests were repeated at admission. Physical examination results indicated normal weight, height, and secondary sexual characteristics. The neurologic examination and visual field test showed mild superior hemianopsia. Paraclinic test results indicated normal CBC, BUN: 9 mg/dl, Cr: 0.8 mg/dl, Na: 148 meq/lit, K: 4 meq/lit, FBS: 97 mg/dl, urine output, 24 hours: 3500 cc, and U/A: SG: 1005. 

The laboratory test results and response to desmopressin acetate nasal spray (DDAP) confirmed the presentation of DI; T4: 4.1 μg/dl; TSH: 0.47 μIU/ml; LH: 0.1 IU/l; FSH: 0.6 IU/l; PRL: 33 ng/ml (dilation 1/10: 1/00); Cortisol: 1 μg/dl; ACTH: 9.4 pg/ml; IGF1: 98 ng/ml. The MRI results four months after the treatment indicated an increased tumor size. The uterus and ovaries had normal conditions based on the ultrasound results and the endometrium’s thickness was 1 mm. We suspected craniopharyngioma, germinoma, and other parasellar tumors, and thus requested for the serum levels of alpha-fetoprotein (AFP) and human chorionic gonadotropin (HCG); that undetectable. Finally, the patient became a candidate for endoscopic transsphenoidal surgery (ETSS).

**Figure 2:**
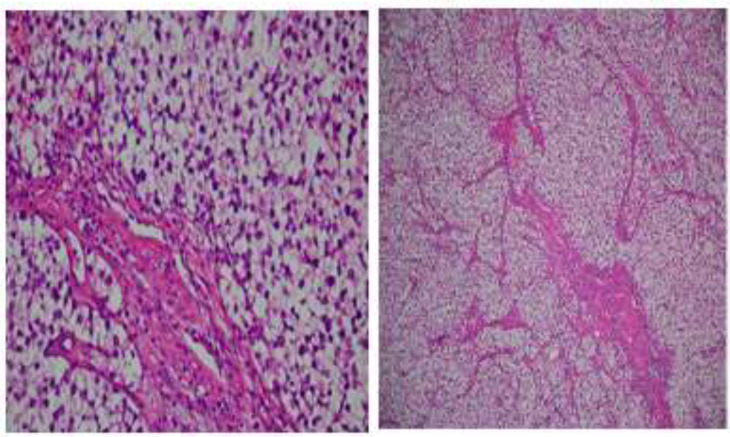
Representative histologic sections of brain biopsy specimens

After the ETSS, the pathology report showed germinoma (figure 2). The immunohistochemical results revealed tumor cells positive for PLAP and CD117 and negative for CK, LCA, and GFAP.

**Figure 3:**
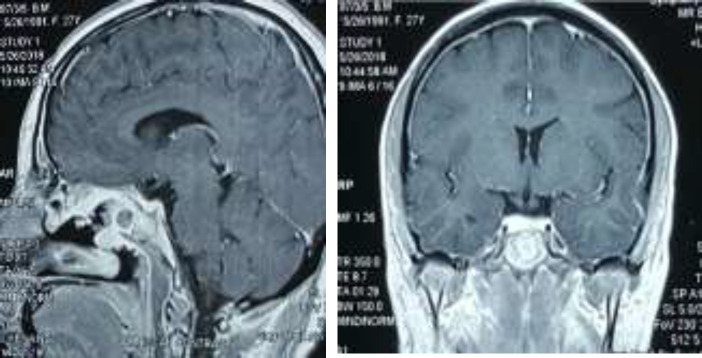
Sagittal (A) and Coronal (B) Post contrast T1W images. No definite residual sellar or suprasellar mass is present. Post-treatment (surgery and radiotherapy) changes depicted including heterogeneous enhancement of sphenoid sinus

In this figure, the hematoxylin and eosin, at a magnification of X100 (A) and X400 (B), show that the cells have clear cytoplasm arrangement in a lobular pattern traversed by thin fibrous septa infiltrated with small mature lymphocytes. The patient received 30 sessions of radiotherapy and hormonal agent therapy, including hydrocortisone, levothyroxine (LT4), estrogen and progesterone, and DDAVP nasal spray. The MRI reports 3-month and 1-year post-operation were normal (figure 3). 

## Discussion

Intracranial germinoma is a rare and hardly distinguishable malignancy. This report showed that germinoma cannot easily be distinguished from other sellar and para sellar mass lesions. Thus, its diagnosis requires several clinical and paraclinical tests. Primary intracranial germ cell tumors (GCTs) are mostly present in the first two decades of life and form 2% to 3% of intracranial neoplasms. Germinomas account for about 60%-70% of all tumors and are more prevalent in Asia (5, 6).Germinomas mainly involve the suprasellar compartment (5, 6). It may be designated as primary intrasellar if both intrasellar and extrasellar components are present (7, 8); however, in our case, the tumor was in the sellar region with suprasellar extension.Suprasellar germinomas are characterized by hypothalamic/pituitary dysfunction, most commonly associated with DI, delayed sexual development, hypopituitarism and/or isolated growth failure (9). We observed endocrine disturbances long before the appearance of neurologic and DI symptoms. Although DI is often the first appearing symptom, it may sometimes develop during the disease course (10, 11). In this case, the delayed diagnosis was because of early endocrine manifestation (menstrual abnormality) without the evidence of DI and cosidering PCOD, hyperprolactinemia (by pituitary stalk impingement), and mismanagement (by the physician).Delay was more likely in patients with a suprasellar tumor (82%) than with a pineal (22%) or bifocal (55%) tumors (12). Although serum levels of β-hCG and AFP were negative, also were needed to be measured in CSF. The clinical findings and elevated concentrations of the tumor markers AFP and β–hCG in CSF indicate the presence of an intracranial germ cell tumor (2). Some studies reported normal serum levels of AFP and HCG. Therefore, specific correlations should be made between the MRI and CT findings (13, 14). Patients with normal β-HCG and/or α-fetoprotein levels are recommended to undergo surgical biopsy for diagnosis; whereas, a biopsy is not necessary for patients with consistent radiologic imaging and elevated serum/CSF levels of β–hCG and AFP (15).These cases can hardly be diagnosed with biochemical and radiological features. On the basis of patient's medical history and disease processing and failure to respond to medication as well as, the symptoms of DI which is unusual in the pituitary adenoma, and highly suggestive of a non-adenomatous lesion, patient candidate for endoscopic transsphenoidal surgery. There are different treatments for germinoma. Radiotherapy is an effective treatment for localized germinomas and low radiation doses with or without chemotherapy is most often a successful treatment (16). As compared to other types of germ-cell tumors, survival rates are higher in patients with germinoma (17). 

In conclusion it is recommended to consider primary intracranial germinoma in the differential diagnosis of pituitary macroadenoma in young adults, mainly when presented with pituitary dysfunction and DI symptoms. The importance of early recognition of this form of presentation is because early radiation and/or chemotherapy can result in neoplasm improvement.
